# Primate dexterous hand movements are controlled by functionally distinct premotoneuronal systems

**DOI:** 10.1126/sciadv.aea1184

**Published:** 2026-02-11

**Authors:** Tomohiko Takei, Tomomichi Oya, Kazuhiko Seki

**Affiliations:** ^1^ Department of Neurophysiology, National Institute of Neuroscience, National Center of Neurology and Psychiatry, Kodaira, Tokyo 187-8502, Japan.; ^2^ Department of Developmental Physiology, National Institute for Physiological Sciences, Okazaki, Aichi 444-8585, Japan.; ^3^ Brain Science Institute, Tamagawa University, Machida, Tokyo 194-8610, Japan.; ^4^ Western Institute for Neuroscience, University of Western Ontario, London, ON N6A 3K7, Canada.; ^5^ Department of Physiology & Pharmacology, University of Western Ontario, London, ON N6A 3K7, Canada.; ^6^ Precursory Research for Embryonic Science and Technology (PRESTO), Japan Science and Technology Agency (JST), Tokyo 102-0081, Japan.

## Abstract

Dexterous hand movements are uniquely developed in primates and indispensable for their daily activities. Traditionally, they were thought to depend primarily on the evolutionarily “newer” direct corticomotoneuronal (CM) pathway. However, recent studies suggest that the “older” indirect corticospinal pathways, mediated by spinal premotor interneurons (PreM-INs), also contribute, highlighting the need to clarify their functional differences. Here, we recorded neuronal activity from PreM-INs and CM cells in macaques during a precision grip task to compare their roles in generating hand muscle activity. Our results show that PreM-INs exert stronger facilitation across a broader set of muscles, promoting synergistic coactivation, whereas CM cells provide more selective facilitation, enabling control of relatively individual muscles. Decomposition analysis further revealed that these systems correspond to different control modes—synergy-based and individual-based control—balancing stability and flexibility. These findings redefine our understanding of primate dexterous hand control as emerging from the cooperative integration of evolutionarily distinct premotoneuronal systems.

## Teaser

Distinct spinal and cortical pathways coordinate muscle synergies and fine control to support primate dexterous hand movements.

## INTRODUCTION

Physiological and anatomical studies have suggested that phylogenetically distinct pathways are involved in the control of dexterous hand movements in primates. A direct corticomotoneuronal (CM) pathway is a phylogenetically newer pathway that has evolved specifically in primates (*1*–*4*). The apparent codevelopment of the CM pathway and dexterous hand function across the primates led us to reason that CM cells play a major role in the control of dexterous hand movements. This idea has been widely accepted and is the fundamental premise for the neural control of dexterous hand movements (*1*, *2*).

In contrast, indirect corticospinal pathways, mediated by spinal premotor interneurons (PreM-INs), are phylogenetically older pathways commonly observed in a diverse range of mammals (*1*, *2*). As these pathways are present in species with less hand dexterity (*1*), their contribution to the control of skilled hand movements has been less appreciated. However, recent studies demonstrated that spinal PreM-INs are involved in controlling skilled hand movements in primates (*5*–*8*). These studies showed that spinal PreM-INs provided postspike facilitation to the hand muscle during a precision grip (*5*). In addition, their activity was correlated with the target muscle activity and grip force (*6*, *7*). These findings suggest that the older PreM-IN pathways also contribute to the control of skilled hand movements in primates, alongside the CM cells.

These findings raise the question of how the multiple premotoneuronal systems contribute to dexterous hand movements in primates: whether they coexist as just redundant structures providing comparable functions or fulfill specialized functions for controlling primate hand movements. In primates, CM cells and PreM-INs are typically identified electrophysiologically on the basis of their postspike facilitation of muscle activity, which reflects their putative direct influence on motoneuron pools (*9*, *10*). Thus, these two pathways provide parallel premotoneuronal inputs to the spinal motoneurons (*11*). Here, we hypothesized that the two systems have different functional roles and that their cooperative integration is essential for the development of dexterous hand movements in primates.

To test this hypothesis, we compared how PreM-INs and CM cells contribute to the generation of hand muscle activity by analyzing spike-triggered averages of muscle activity during a precision grip task. Our findings showed that a PreM-IN exhibited postspike facilitation on a larger number of hand muscles, and its firing activity had a higher correlation with the target muscle activity than was measured with a CM cell. Through the convolution of postspike effects with neuronal firing activity, we demonstrated that PreM-INs have a greater contribution than CM cells to the generation of hand muscle activity during the precision grip task. Furthermore, decomposition analyses of muscle activity demonstrated that PreM-IN and CM cell activity corresponds to synergy- and individual-based control balancing stability and flexibility in dexterous hand movements.

These results suggest that the two systems have specialized functions in the control of primate dexterous hand movement. Specifically, PreM-INs effectively produce a gross pattern of muscle activity through synergistic activation. In contrast, CM cells add a finer and more selective tuning of relatively individual muscles. The cooperative integration of functionally distinct premotoneuronal systems, synergistic and fine-tuning, may underlie the development of dexterous hand function in primates.

## RESULTS

### PreM-INs coactivate a broader set of muscles, whereas CM cells have more selective facilitation

**Fig. 1.**
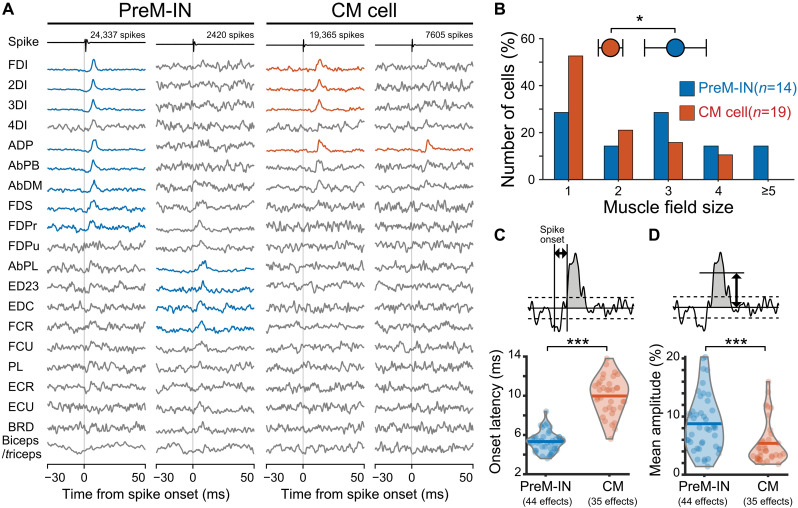
Comparison of postspike facilitations of PreM-INs and CM cells. (**A**) Spike-triggered averages of rectified EMG signals by example PreM-INs (left) and CM cells (right). Spike-triggered averages with significant postspike facilitations are indicated in blue (PreM-IN) or red (CM cell). Top row, spike waveform. Bottom rows, EMG signals. EMG abbreviations can be found in Materials and Methods. (**B**) Comparison of muscle field size. Circles, means. Error bars, standard error of means. *t* test, *P < 0.05. Bottom bars, histogram of the muscle field size of PreM-INs (blue, *n* = 14) and CM cells (red, *n* = 19). (**C** and **D**) Comparison of onset latency (C) and mean amplitude increase (D) of postspike facilitation (shaded area). Violin plots indicate the distribution of each measure. Each filled dot indicates a postspike effect of PreM-INs (blue, *n* = 44) and CM cells (red, *n* = 35). Horizontal bars, means. *t* test, ***P < 0.001.

To test the difference in the output effects of PreM-INs and CM cells on hand muscle activity, we examined the postspike facilitation of PreM-INs and CM cells. We recorded single-unit activity from the cervical spinal cord or hand region of the primary motor cortex in two monkeys performing a precision grip task (fig. S1). We also recorded electromyogram (EMG) activity from 20 hand and arm muscles using chronically implanted wire electrodes, allowing a comprehensive assessment of the output effects of both PreM-INs and CM cells. Using a spike-triggered average of EMGs, we identified postspike effects, which are considered to represent putative direct effects on spinal motoneuron pools (*9*, *12*, *13*). Of the 211 spinal and 234 cortical neurons recorded from two monkeys, 14 and 19 neurons produced 44 and 35 postspike facilitations and were identified as PreM-INs and CM cells, respectively. From the spinal cord of one monkey (monkey S), only one case of postspike suppression was detected. Because the overall sample size of postspike suppressions was small (five PreM-INs and two CM cells), these effects were excluded from the present analysis. Consequently, all spinal data presented in this study were obtained from one monkey (monkey E). Figure 1A shows examples of postspike facilitation produced by PreM-INs and CM cells. A substantial population of neurons in both groups exhibited postspike facilitation on more than one hand muscle, commonly referred to as the muscle field (fig. S2 for all neuron data) (*9*, *14*). We compared the size of the muscle field, defined as the number of muscles facilitated by each neuron, and found that the muscle field was larger for PreM-INs than for CM cells (3.1 ± 2.3 versus 1.8 ± 1.1, *P* < 0.05, *t* test, *t*_[31]_ = 2.16; Fig. 1B and fig. S4A for each animal), indicating that PreM-INs coactivated a larger number of hand muscles.

Next, we compared the onset latency and size of the postspike facilitation between the groups (Fig. 1, C and D, and fig. S4, B and C). The onset latency was shorter for PreM-INs than for CM cells (5.3 ± 1.0 versus 10.0 ± 1.9 ms, *P* < 0.001, *t* test, *t*_[48.85]_ = −12.85), which was expected because of the relative proximity of muscles to the spinal cord compared with the cerebral cortex. In addition, the postspike facilitation size was larger in PreM-INs than in CM cells (8.8 ± 4.9% versus 5.4 ± 3.7%, *P* < 0.001, *t* test, *t*_[76.79]_ = 3.49), particularly in one monkey (monkey S; fig. S4C). These results indicate that PreM-INs coactivate a larger number of muscles with a shorter latency and greater amplitude, whereas CM cells have postspike effects on a limited set of muscles with a longer latency and smaller amplitude.

### PreM-INs show target muscle–like activity, whereas CM cells show a diverse activity pattern

**Fig. 2.**
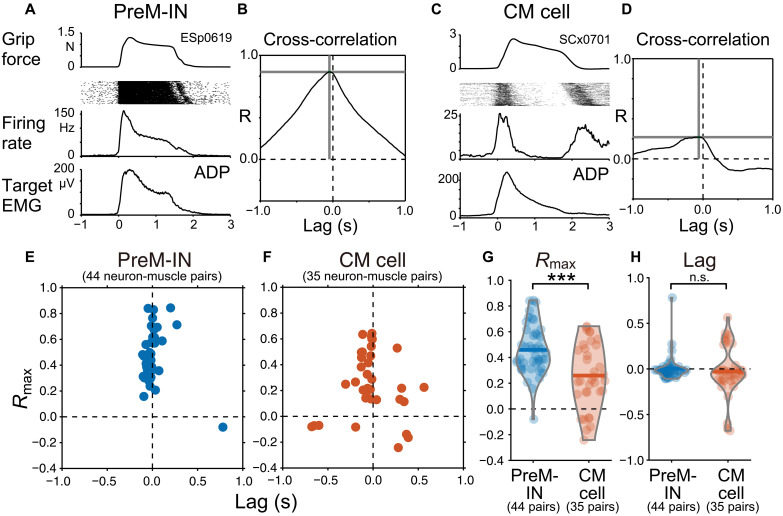
Temporal correlation with target muscle activity. (**A**) Activity profile of an example PreM-IN (ESp0619) and one of its target muscles (ADP) showing (from top to bottom) averaged grip force, raster of spikes, averaged firing rate, and averaged EMG across trials (211 trials). (**B**) Cross-correlogram between neural and muscle activity. The peak correlation value (*R*_max_) and its lag are indicated by horizontal and vertical gray lines, respectively. (**C** and **D**) Same format but for an example CM cell (SCx0701) and target muscle (ADP) pair (380 trials). (**E** and **F**) Distribution of *R*_max_ and lag for PreM-INs (E) and CM cells (F). (**G** and **H**) Comparison of *R*_max_ (G) and lag (H). Each filled dot indicates a neuron-muscle pair with PreM-INs (blue, *n* = 44) and CM cells (red, *n* = 35). Horizontal bars, means. *t* test, ***P < 0.001. n.s., nonsignificant.

Given the faster and stronger facilitation observed in PreM-INs to the target muscles, we anticipated that PreM-IN activity would exhibit a closer temporal relationship with the target muscle activity than would be observed with CM cells. To test this, we computed the cross-correlation between the neural firing activity and the target muscle. For neurons with multiple target muscles, the correlation was calculated separately for each neuron-muscle pair. Figure 2 (A to D) shows examples of the cross-correlations between the activity of a PreM-IN and that of a CM cell with one of their target muscles. The cross-correlogram demonstrated that the PreM-IN activity had a high correlation value (*R*_max_ = 0.84) with a peak lag of −4.8 ms, indicating that the PreM-IN activity preceded the target muscle activity with a short latency (Fig. 2B). The cross-correlation of a CM cell showed a lower correlation value (*R*_max_ = 0.22), and it did not exhibit a clear peak preceding the target muscle activity (Fig. 2D). Figure 2 (E and F) shows the distribution of *R*_max_ and peak lag for neuron-muscle pairs of PreM-INs and CM cells. On average, the PreM-INs had a higher correlation value (0.46 ± 0.19), with the peak lag clustered at around zero (4.6 ± 0.14 ms), whereas CM cells had a lower correlation value (0.26 ± 0.25, *P* < 0.001, *t* test, *t*_[62.11]_ = 3.86; Fig. 2G and fig. S4D), and the lag was broadly distributed (−28.5 ± 274.6 ms, *F* test for equal variances, *P* < 0.001, *F*_43,34_ = 0.26; Fig. 2H and fig. S4E). These results suggest that PreM-INs have more target muscle–like activity, whereas CM cells have diverse temporal relations with target muscles.

### PreM-INs have a greater contribution to the generation of hand muscle activity

**Fig. 3.**
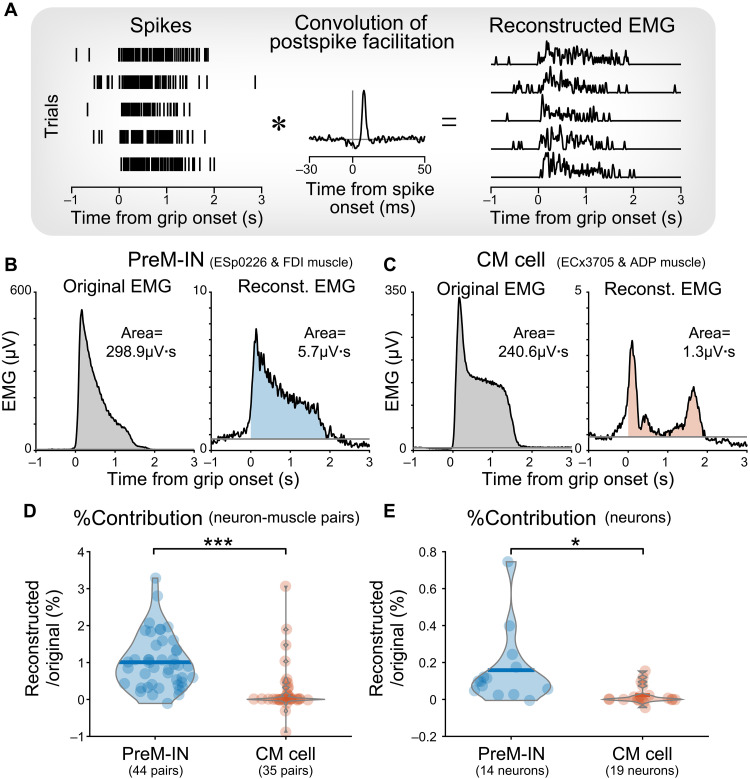
Reconstruction of EMG from single neuron activity. (**A**) Schematic illustration of the procedure to reconstruct EMG by single neuron activity. Muscle activity generated by a single neuron was estimated by convolving spike firing and a waveform of a postspike effect obtained from spike-triggered averaging (STA). Left, spike raster from five example trials. Center, example of spike-triggered average. Right, reconstructed EMG. The asterisk (*) denotes the convolution operation. (**B** and **C**) Exemplar data of the original and reconstructed muscle activity [FDI in (B) and ADP in (C)] by a single PreM-IN (ESp0226) (B) and CM cell (ECx3705) (C). The shaded areas indicate the muscle activity in the analytical time window (0 to 2 s from grip onset). (**D**) Relative contribution of single neuron activity to the generation of a single target muscle activity. Each filled dot indicates a neuron-muscle pair with PreM-INs (blue, *n* = 44) and CM cells (red, *n* = 35). (**E**) Aggregated contribution of single neuron activity to the activity of entire recorded muscles. Each filled dot indicates a single neuron, PreM-INs (blue, *n* = 14), and CM cells (red, *n* = 19). Horizontal bars, means. *t* test, *P < 0.05. ***P < 0.001.

To compare the contribution of PreM-INs and CM cells in the generation of hand muscle activity, we estimated the EMG activity generated by individual neurons and compared their relative amplitude to the original EMG activity. We reconstructed EMG activity produced by a single neuron by convolving a waveform of postspike facilitation with the firing activity of the triggering neuron (Fig. 3A). Examples of reconstructed EMG of single PreM-IN and CM cells are shown in Fig. 3 (B and C). The reconstructed EMG of a PreM-IN had a temporal profile similar to that of the original EMG, reflecting the high temporal correlation between the neurons and target muscles (Fig. 3B). To quantify the relative contribution of individual neurons, we calculated a ratio of the area under the EMG profiles during a grip period (reconstructed/original, %). The example PreM-IN contributed 1.9% (5.7 of 298.9 μV·s) to the original EMG activity, whereas the reconstructed EMG by a CM cell had a smaller contribution of 0.6% (1.3 of 240.6 μV·s) (Fig. 3C). On average, PreM-INs contributed approximately four times more to target muscle activity generation than CM cells (1.01 ± 0.73% versus 0.25 ± 0.68%, *P* < 0.001, *t* test, *t*_[74.81]_ = 4.75; Fig. 3D and fig. S4F).

Given that PreM-INs had a larger muscle field (Fig. 1), their relative contribution to generating the activity of muscles could be magnified when considering the activity of all muscles required for performing the task. To investigate the contribution of individual neurons to the generation of overall muscle activity, we calculated the aggregate contribution to all recorded muscles, including the muscles with or without postspike facilitations, for each individual neuron (Fig. 3E). We found that the relative contribution of PreM-INs (0.16 ± 0.20%) was eight times greater than that of CM cells (0.02 ± 0.05%, *P* < 0.05, *t* test, *t*_[14.24]_ = 2.49; Fig. 3E and fig. S4G). These results suggest that a PreM-IN contributes to the formation of gross activity over multiple muscles during precision grip, whereas a CM cell provides fine-tuning of the muscle activity in a more specific manner.

### PreM-INs and CM cells are involved in synergy- and individual-based control of hand muscles

**Fig. 4.**
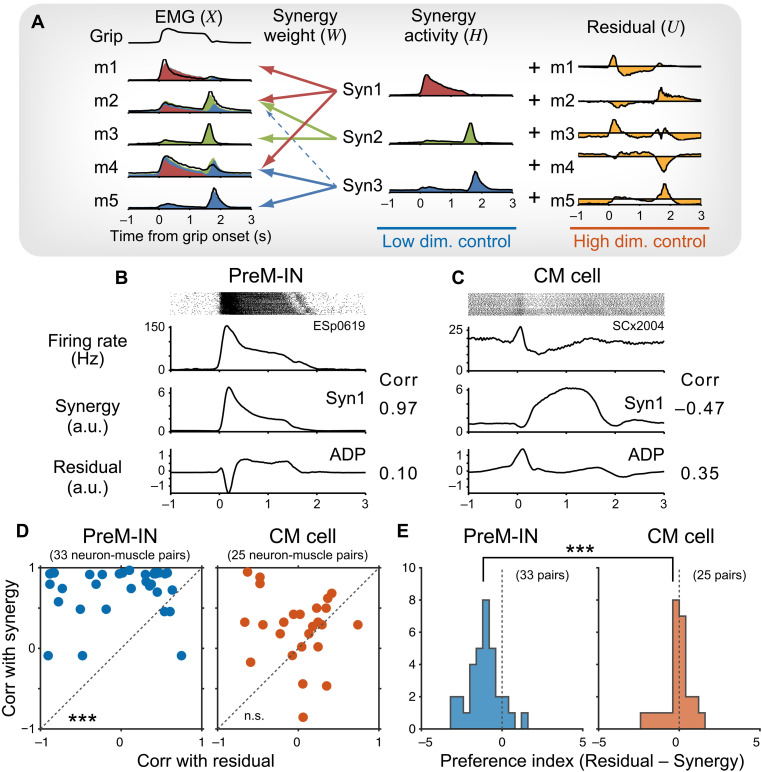
Comparison with a muscle synergy model. (**A**) Schematic illustration of a muscle synergy model. A linear combination of a small number of muscle synergies explains the majority (~90%) of variance of original EMG (m1 to m5), and residuals (~10%) are added to the individual muscles. a.u., arbitrary units. (**B** and **C**) Firing activity of exemplar neurons of PreM-INs (ESp0619) (B) and CM cells (SCx2004) (C) and their preferred synergy (Syn1) and residual component of one of their target muscle’s activity (ADP). The numbers on the right side indicate the correlation coefficient calculated between neural activity and synergy or residual components. (**D**) Comparison of correlation of PreM-IN (left) or CM cell activity (right) with the preferred synergy and residual of the target muscle activity. Each dot indicates a neuron-muscle pair with PreM-INs (blue, *n* = 33) and CM cells (red, *n* = 25). Dashed lines indicate equality. Paired *t* test after Fisher’s *z* transformation, ***P < 0.001. (**E**) Comparison of the preference index between PreM-INs (left, *n* = 33) and CM cells (right, *n* = 25). *t* test, ***P < 0.001.

Our results demonstrated distinct properties of PreM-INs and CM cells in controlling hand muscle activity during a precision grip task. On the basis of these observations, we proposed a functional hypothesis that the two pathways play complementary roles in the control of hand muscles: PreM-INs primarily contribute to generating gross muscle activity through the synergistic activation of multiple muscles, whereas CM cells provide fine-tuning of relatively individual target muscles. To translate this functional hypothesis into a testable prediction, we compared the PreM-IN and CM cell activity with a muscle synergy model (*15*). The muscle synergy model assumes that a hand muscle’s activity is generated through a linear combination of a small number of low-dimensional modules referred to as muscle synergies. Conventionally, muscle synergies are used to explain a majority (~90%) of the variance of the overall hand muscle activity (*15*). However, a considerable portion of variance (~10%) remains unexplained as the “residuals” and is often ignored as noise. Recent studies, however, highlighted the unexpected complexity of primate hand movements and suggested that the higher-dimensional variance, not captured by low-dimensional components, may incorporate substantial volitional control (*16*–*18*). Therefore, we modeled hand muscle activity during a precision grip as a combination of lower-dimensional control by muscle synergies and higher-dimensional control represented in the residuals (Fig. 4A). Our hypothesis predicted that the PreM-IN activity would correlate primarily with the muscle synergy, whereas the CM cell activity would correlate with the residual of the target muscle activity in addition to muscle synergy.

Dimensional reduction analysis showed that a small number of muscle synergies (three and four in monkeys E and S, respectively) explained ~90% of the variance in muscle activity, as shown in a previous study (fig. S3). Figure 4B shows an example of the correlation between PreM-IN activity and the preferred muscle synergy, whose synergy weights showed the highest similarity with the neuron’s muscle field (*15*) and with the residual of one of the target muscles. This PreM-IN showed a higher correlation with the preferred synergy and lower correlation with the target muscle residual (Fig. 4B). In contrast, an example CM cell showed a higher correlation with the residual and lower correlation with the preferred synergy (Fig. 4C). Figure 4D provides a comparison of the correlations between each neuron’s activity with the preferred synergy and the residual of the target muscle activity. For the neurons with multiple target muscles, the correlation was calculated separately for each neuron-muscle pair. On average, a PreM-IN had a higher correlation with synergy than with residual (0.73 ± 0.31 versus −0.04 ± 0.56, *P* < 0.001, paired *t* test with *z* transformation, *t*_[32]_ = 7.76; Fig. 4D and fig. S4H), whereas a CM cell was more evenly correlated with both the synergy and residual (0.25 ± 0.43 versus −0.00 ± 0.37, *P* = 0.063, paired *t* test with *z* transformation, *t*_[24]_ = 1.95; Fig. 4D and fig. S4, I and J). To assess the preference of each neuron, we computed a preference index (Fig. 4E) and found that PreM-INs showed a higher preference for synergy, whereas CM cells exhibited a more balanced preference for synergy and residual (−1.28 ± 0.95 versus −0.33 ± 0.84, *P* < 0.001, *t* test, *t*_[54.67]_ = −4.07; Fig. 4E and fig. S4, K to M). These results supported our hypothesis that PreM-INs primarily contribute to synergy-based control, whereas CM cells provide additional fine-tuning of the target muscle activity.

## DISCUSSION

In this study, we demonstrated distinct properties of PreM-INs and CM cells in the control of skilled hand movements in primates. PreM-INs exhibited a synergistic coactivation of a larger number of muscles, suggesting the generation of gross hand muscle activity. In contrast, CM cells showed more selective control of relatively individual muscles, suggesting fine-tuning of target muscles. The low-dimensional control of muscles via muscle synergies, as observed in PreM-INs, has been previously associated with enhancing the efficiency and robustness of motor control (*19*–*24*). In contrast, fine-tuning of relatively individual muscle activity is essential for achieving fractionated control of hand muscles (*14*, *25*) and independent finger movements (*26*, *27*). Our results provide direct physiological evidence that these two aspects of hand motor control are mediated by distinct premotoneuronal systems with different properties. Given that both systems come into play during skilled hand movement, such as precision grip, it is conceivable that the coordination of the efficient (synergistic) and flexible (fractionated) control systems is essential for achieving the substantial hand dexterity in primates.

Over the past 40 years, studies have consistently demonstrated the central role of CM cells in voluntary control of skilled hand movements, including precision grip (*14*, *25*, *28*–*30*), relatively independent finger movements (*13*, *31*, *32*), and wrist movements (*9*, *33*, *34*). Notably, these studies have demonstrated that CM activity does not simply reflect target muscle activity. For example, Muir and Lemon (*28*) showed that a CM cell was selectively activated during a precision grip but was deactivated during a power grip, even though the target muscle remained active. Similarly, Griffin *et al.* (*33*) showed that the relationship between CM cells and target muscles was broadly distributed. Our findings suggest that this unexplained nature of CM cells reflects their contribution to high-dimensional control of hand muscles, which was not captured by the low-dimensional control responsible for a majority of variance (Fig. 4A). Given that CM cells have a smaller contribution to the generation of muscle activity (Fig. 3, D and E), it is plausible that their role is to provide fine-tuning of target muscles on top of the lower-dimensional synergistic control. In contrast, the phylogenetically older PreM-INs play a more substantial role in generating gross hand muscle activity (Fig. 3, D and E) and are specifically correlated with the lower-dimensional control (Fig. 4A). These results suggest that over the course of primate evolution, the CM system did not replace or override the function of the older systems (*2*) but rather added a newer layer of control that enhances the flexibility of primate hand motor control.

Although there is a relative difference between the CM cells and PreM-INs, there is also a large overlap in their functional properties. This overlap is evident in the distribution of muscle field size (Fig. 1B), temporal correlation with target muscle activity (Fig. 2G), and the preference index of muscle synergy and residuals (Fig. 4E). These observations suggest that the functional differentiation of these two systems is not mutually exclusive but rather is gradual and characterized by considerable overlap. It has been demonstrated that corticospinal terminals are distributed within the intermediate zone at birth and then gradually extend into the ventral horn over the first 5 months of postnatal development in the monkey spinal cord (*35*). This anatomical shift suggests that the corticospinal tract progressively incorporates the indirect connections and direct CM projections, leading to the gradual overlap of functional properties between the two systems.

Another factor determining the relative contributions of PreM-INs and CM cells is the number of neurons recruited. Although the relative contribution of individual neurons was higher for PreM-INs than CM cells (Fig. 3), the overall contribution could be greater for CM cells if a larger number of CM cells are recruited. To our knowledge, there are currently no comprehensive anatomical data comparing the total number of these two neuronal populations. Future studies using molecular or genetic tools to identify and manipulate each population may allow for a quantitative comparison of their overall contributions.

A previous comparison of CM cells and PreM-INs in the context of voluntary wrist movements demonstrated that spinal PreM-INs have a smaller muscle field than the CM cell (*10*), which conflicts with our present findings. Several reasons might account for this discrepancy. First, wrist movements typically involve the reciprocal activation of antagonist muscles, whereas hand grasping often requires the coactivation of antagonistic muscles (*36*–*38*). Notably, during wrist movements, a substantial number of CM cells showed a reciprocal muscle field, characterized by both excitatory and inhibitory postspike effects on antagonistic muscles at the same time (*39*), a phenomenon not observed in our present study. This suggests that different control strategies are used during wrist movements (reciprocal) and grasping (coactivating). Second, the previous study compared CM cells and PreM-INs on the basis of the data compiled from different experiments, which used different criteria to identify the postspike effects (*9*, *10*, *40*). In the present study, we used consistent analytical procedures for both cortical and spinal recordings in the same animals, enabling us to directly compare the detailed physiological properties.

Spinal PreM-INs receive convergent inputs from various descending tracts (*41*) and peripheral afferents (*42*). This indicates that the lower-dimensional control mediated by the spinal PreM-INs may serve as a fundamental platform for both voluntary and reflexive hand functions in primates. In addition, classical anatomical studies in macaques have demonstrated that some CM axons give off collaterals within the intermediate zone where spinal PreM-INs reside (*43*). Thus, it is possible that CM cells may also influence spinal motoneurons indirectly through PreM-INs, in addition to their direct connection. In the present study, however, neurons were classified on the basis of postspike effects with short onset latency and narrow peak width [peak width at half-maximum (PWHM) <7 ms], which preferentially captures putative direct premotoneuronal coupling (*9*, *12*, *13*). If CM outputs were mainly conveyed via PreM-INs, the muscle fields of CM cells should be larger than those of PreM-INs owing to the additional divergence through PreM-INs. However, CM cells exhibited smaller muscle fields than PreM-INs (Fig. 1B), suggesting that any collateral-mediated influence, although possible, is unlikely to account for the distinct output properties observed. These considerations support the interpretation that CM cells and PreM-INs provide functionally distinct, largely parallel inputs to spinal motoneurons.

One limitation of our dataset is that all spinal data reported in this study were obtained from a single monkey (monkey E). In another monkey (monkey S), the instability of the spinal recording chamber restricted neural recording to only four sessions. During that period, we recorded a total of 12 units, including one inhibitory PreM-IN. This proportion was nearly identical to that observed in monkey E (19 PreM-INs identified among 199 recorded units), suggesting that the low yield in monkey S was primarily due to technical difficulties in maintaining the recording chamber rather than differences in neuronal properties. Future studies using more stable and advanced high-density spinal recording techniques will be required to overcome these limitations and to provide a more comprehensive dataset.

## MATERIALS AND METHODS

### Dataset

The datasets used in the present study were obtained from two male macaque monkeys (monkey E: *Macaca mulatta*, 5.6 kg at the age of 5; monkey S: *Macaca fuscata*, 9.0 kg at the age of 8). The spinal recording dataset from monkey E was the subject of previous reports (*5*–*7*, *15*). The rest of the datasets (cortical recordings from monkeys E and S and spinal recordings from monkey S) were newly collected for this study. Only a postspike suppression was identified in the spinal recording of monkey S, which was excluded from the present report. Therefore, all spinal data presented here were obtained from monkey E. All procedures were approved by the Animal Research Committee at the National Institute for Physiological Sciences, Aichi, Japan (study approval nos. A16-85-51, A17-76-52, A18-76-47, 07A167, and 08A095) and the experimental animal committee of the National Institute of Neuroscience, Tokyo, Japan (study approval nos. 2009-13 and 2012-004).

### Behavioral task

The monkeys were trained to grip spring-loaded levers with their left index finger and thumb (precision grip task; fig. S1A) (*5*, *44*). Lever positions were displayed on a computer screen as cursors, and monkeys were required to track targets in a step-tracking manner. Each trial comprised a rest period (1.0 to 2.0 s), lever grip, lever hold (1.0 to 2.0 s), and lever release. Successful completion of a trial was rewarded with a drop of apple sauce (fig. S1D). The force required to reach the target positions was adjusted independently for the index finger and thumb of each monkey.

### Surgical procedures

After the task training was completed, we performed surgeries to implant head restraints, EMG wires, and recording chambers under isoflurane or sevoflurane anesthesia and aseptic conditions. For EMG recordings, we subcutaneously implanted pairs of stainless steel wires (AS 631, Cooner Wire, Chatsworth, CA) in the forelimb muscles, including intrinsic hand muscles [first, second, third, and fourth dorsal interosseous (FDI, 2DI, 3DI, and 4DI, respectively); adductor pollicis (ADP); abductor pollicis brevis (AbPB); and abductor digiti minimi (AbDM)], extrinsic hand flexors [flexor digitorum superficialis (FDS) and radial and ulnar parts of the flexor digitorum profundus (FDPr and FDPu, respectively)], wrist flexors [flexor carpi radialis (FCR) and flexor carpi ulnaris (FCU)], extrinsic hand extensors [abductor pollicis longus (AbPL), extensor digitorum-2,3 (ED23), and extensor digitorum communis (EDC)], a wrist extensor [palmaris longus (PL), extensor carpi radialis (ECR), and extensor carpi ulnaris (ECU)], and elbow muscles [brachioradialis (BRD, biceps brachii (biceps), and triceps]. The wire electrodes were passed subcutaneously from the junction between the head implant and the skin to one of the incisions made over the target muscles. The wires and connectors were then fixed to the head implant with dental acrylic (*45*).

For spinal recordings, we implanted a recording chamber on the cervical vertebrae (C4 to C7) of monkeys, where unilateral laminectomy was performed on the ipsilateral side of the used hand (fig. S1B). For cortical recordings, we implanted a recording chamber (a circular cylinder with a 50-mm diameter) over a craniotomy covering a cortical area, including the hand representation of pre- and postcentral gyri on the contralateral side of the used hand (fig. S1C).

### Data recordings

While the monkey was performing the precision grip task, we recorded single-unit activity from cervical 6 (C6)-thoracic 1 (T1) segments or from the hand area of the primary motor cortex with a tungsten or Elgiloy microelectrode (impedance of 1 to 2 MΩ at 1 kHz) with a hydraulic microdrive (MO-951, Narishige Scientific Instrument, Tokyo, Japan). We determined the recording sites with the aid of positions of vertebral segments for the spinal recordings and the sulcal structures and intercortical electrical microstimulation (300 Hz, 10 pulses, <20 μA) for the cortical recordings. Action potential timing was detected online using a spike-sorting device (MSD; Alpha Omega Engineering, Nof HaGalil, Israel), and spike-triggered averaging of rectified EMG signals was monitored during recording. Neural signals and spike times were digitized at 25 kHz. EMGs were bandpass filtered (5 Hz to 3 kHz) and sampled at 5 kHz. Simultaneously with the neural and EMG signals, we recorded grip force and behavioral event timing at 1 kHz (fig. S1E).

### Identification of postspike effects of spinal PreM-INs and CM cells

Detailed analyses of the postspike effects of spinal neurons (*5*) and their activity (*6*, *7*) were reported previously. All analyses were performed offline using MATLAB (MathWorks). The spike-triggered average of the rectified EMG was computed to identify postspike effects of the spinal or cortical neurons on the recorded EMGs (*5*–*7*, *15*). We analyzed only neurons with ≥2000 recorded spikes. Spike-triggered averages were compiled by averaging segments of rectified EMG activity from 30 ms before to 50 ms after each trigger. Spikes were accepted as triggers only if the root mean square value of the EMG from 30 ms before to 50 ms after the spike was greater than 1.25 times the root mean square noise level in that EMG channel. The spike-triggered average was smoothed with a flat five-point finite impulse response filter. The baseline trend was subtracted using the incremented-shifted averages method (*46*), and significant spike-triggered average effects were identified with multiple-fragment statistical analysis (*32*). The test window was set to 3 to 15 ms after the spinal neuron spike and 6 to 16 ms after the cortical neuron spike (*13*).

Potential cross-talk between recorded EMGs was evaluated by combining a cross-correlation method (*14*) and the third-order differentiation (*47*), and spike-triggered average effects potentially resulting from cross-talk between EMG recordings were eliminated from the present dataset. To distinguish the postspike effects from the synchrony effects (*13*), we measured the onset latency and PWHM; effects with an onset latency >3.5 ms for spinal neurons or >5 ms for cortical neurons and a PWHM <7 ms were identified as postspike effects (*5*, *13*).

A significant short-latency facilitation with a narrow peak width was taken to indicate a putative direct influence on motoneuron pools, following established electrophysiological criteria (*9*, *12*, *13*). Although this approach cannot entirely exclude disynaptic contributions, it is widely used to operationally define CM cells and PreM-INs. The neurons that showed postspike effects on at least one muscle were identified as PreM-INs or CM cells. If spinal neurons showed a large “motor unit” signature in the spike-triggered average of the unrectified EMG with only 50 spikes (*48*), they were identified as putative motoneurons and excluded from the dataset. In our dataset, only two spinal neurons met the criteria for putative motoneurons and were excluded from the present study.

The mean percent increase in a post-spike effect was quantified by averaging the postspike effect amplitude from the onset to offset, subtracting the baseline mean, and then dividing the result by the baseline mean and multiplying by 100. The muscle field was defined as a distribution of postspike effects produced by a single PreM-IN or CM cell and was represented as a binary vector indicating the presence or absence of postspike effects.

### Temporal correlation between firing activity and target muscle activity

To quantify the temporal correlation between neuronal activity and target muscle activity, we calculated cross-correlations between the neuronal and muscle activities. First, the neuron instantaneous firing rate [IFR(*t*)] was calculated as the inverse of the interspike interval: IFR(*t*) = 1/(*t*_*i*+1_ − *t_i_*), for *t_i_* < *t* < *t*_*i*+1_, where *t_i_* is the time of the *i*th spike. The instantaneous firing rate was then low-pass filtered (second order, Butterworth, cutoff of 20 Hz in forward and backward directions) and downsampled to 1 kHz. Rectified EMGs were also low-pass filtered (second order, Butterworth, cutoff of 20 Hz in forward and backward directions) and downsampled to 1 kHz. Continuously recorded 90-s data points, which contained ~10 successive trials, were used to calculate the cross-correlation.

### Reconstruction of EMG signals from the postspike effect of spinal interneurons

To estimate the contribution of single premotor neurons to the generation of muscle activity, we reconstructed the EMG signal by convolving neural firing activity and a postspike effect waveform. We used the spike-triggered average waveform, whose baseline trend was subtracted using the incremented-shifted averages method (*46*), as a kernel. Because the waveform contained the EMG signal from 30 ms before to 50 ms after spike firing, we padded zeros for 20 ms before the waveform to center the spike timing in the kernel. Next, we converted the spike timing signal to a continuous binary signal (0 or 1) at a 1-kHz sampling rate. Then, we convolved the kernel with a spike count signal to reconstruct the EMG. Note that EMG signals were not normalized and were in voltage (μV) units to compare the original and reconstructed signals. To evaluate the relative contribution of each premotor neuron, we averaged the original or reconstructed EMG signal, segmented from −1 to 3 s from the grip onset; measured the area during the grip period (0 to 2 s from the grip onset); and calculated the ratio of the area of the reconstructed EMG to that of the original EMG as an index of the relative contribution (% contribution).

### Extraction of muscle synergy

For the muscle synergy analysis, we selected 12 muscles to maintain consistency with our previous study (FDI, ADP, AbPB, AbDM, FDS, FDPr, FDPu, FCR, FCU, ED23, EDC, and ECU) (*15*). Muscle synergies (synergy weights and synergy activity) were estimated from the EMGs using nonnegative matrix factorization (NMF) (*49*). First, we removed electrical cross-talk between EMG signals by applying blind-signal separation for the third-order differentiated EMG signals (*47*). EMG signals were downsampled to 1 kHz and differentiated three times. Then, blind-signal separation was used with the preprocessed signals to remove the nonphysiological electrical cross-talk. After the separation, the signals were third-order integrated to restore the original waveforms.

NMF was applied to the processed EMG signals. We used 8-min-long continuously recorded EMG signals. The separated EMGs were high-pass filtered (cutoff, 50 Hz), rectified, low-pass filtered (20 Hz), linearly smoothed (100 time points), and downsampled to 100 Hz. The amplitude of EMGs was normalized to set the mean amplitude to 1.

The NMF algorithm was initialized with random weight and activity matrices, which were drawn from a uniform distribution between 0 and 1. Values of these matrices were iteratively updated using the multiplicative rule until a change of EMG reconstruction *R*^2^ < 0.01% in 20 consecutive iterations. Because the solutions for the synergies and their coefficients could fall into a local minimum, we repeated the synergy extraction 10 times from different initial values. We selected the synergies that showed the highest *R*^2^ for further analyses.

To identify the number of muscle synergies, we successively increased the number of synergies extracted from one to the number of muscles recorded (fig. S3). The *R*^2^ values were obtained with fourfold cross-validation by first extracting synergies from three of four 2-min-long datasets (the training sets) and then fitting the extracted synergies to the other unused quarter (the testing set). We averaged *R*^2^ values of the fourfold testing set and plotted them against the number of synergies extracted (fig. S3, A and B). The number of synergies was defined as the point at which *R*^2^ was the closest to 0.9 (*R*^2^ = 0.89 and 0.91 with three and four synergies for monkeys E and S, respectively). The synergy weights and synergy activity were normalized to set the mean value of synergy activity to 1.

### Correlations of neural activity with muscle synergies and residuals

To compare the functional difference between PreM-INs and CM cells, we modeled hand muscle activity as a combination of lower- and higher-dimensional control, represented by muscle synergies and residuals, respectively. We calculated residuals by reconstructing the EMGs only with a linear combination of muscle synergies. As a definition, the muscle synergies account for ~90% of the variance of original EMGs. Then, we subtracted the reconstructed EMGs from the original EMGs to define residuals, which account for ~10% of the variance of the original EMGs.

The correlation of the temporal profiles of premotor neuronal activity with synergy activity or residuals was quantified with the response profile aligned to the grip onset. Grip onset was defined as the time at which the rate of change of the total grip force exceeded 2 N/s. The response averages of instantaneous firing rate or synergy activity were compiled by aligning with the grip onset (from 1 s before to 3 s after the grip onset) and averaging across trials. The correlation of the neural activity with a muscle synergy or a residual was quantified as Pearson’s correlation coefficient (*R*) between the two waveforms.

For each neuron-target muscle pair, we compared the neural correlation with the preferred muscle synergy and the residual of the target muscle. The preferred muscle synergy was defined with the similarity in the neuron’s muscle field and synergy weight, quantified as an absolute value of the cosine angle between the two vectors (muscle field and synergy weight) in muscle dimension (*n* = 12) (*15*). This index ranged from 0 to 1, where 1 indicates perfect matching and 0 indicates no correlation. After we computed the similarity for all muscle synergies (three and four for monkeys E and S, respectively), we defined the muscle synergy with the highest similarity as a preferred synergy of the premotor neuron. To compare the waveform correlation of neural firing with the preferred synergy and the residual of target muscles in each neuron group (PreM-INs or CM cells), we used a paired *t* test after the correlation coefficient was applied to Fisher’s *z* transformation.

The preference index of each premotor neuron for the synergy or residual of the target muscle was calculated by subtracting the *z*-transformed correlation coefficient [atanh(*R*)] with the synergy from that of the residual (Fig. 4E). Therefore, a higher value indicates a higher preference for the residual, whereas a lower value indicates a higher preference for the synergy. The Kolmogorov-Smirnov test showed that the distributions of preference index of both populations did not deviate from normality (PreM-INs: *D* = 0.085, *P* = 0.96; CM cells: *D* = 0.21, *P* = 0.19), and an *F* test confirmed the equality of variances between the two groups (*F*_32,24_ = 1.29, *P* = 0.52). Therefore, we used a *t* test for the comparison of the preference index between the two groups.
