# Supplementary material for: Primate dexterous hand movements are controlled by functionally distinct premotoneuronal systems
Source: Sci Adv. 2026 Feb 11;12(7):eaea1184. doi: 10.1126/sciadv.aea1184 (PMC12893230; doi:10.1126/sciadv.aea1184)
Supplement: Supplementary file 1 — Figs. S1 to S4 [file sciadv.aea1184_sm.pdf]

Science Advances
AAAS

Supplementary Materials for

Primate dexterous hand movements are controlled by functionally distinct premotoneuronal systems

Tomohiko Takei *et al.*

Corresponding author: Tomohiko Takei, takei@lab.tamagawa.ac.jp; Kazuhiko Seki, seki@ncnp.go.jp

Sci. Adv. **12**, eaea1184 (2026)
DOI: 10.1126/sciadv.aea1184

This PDF file includes:

Figs. S1 to S4

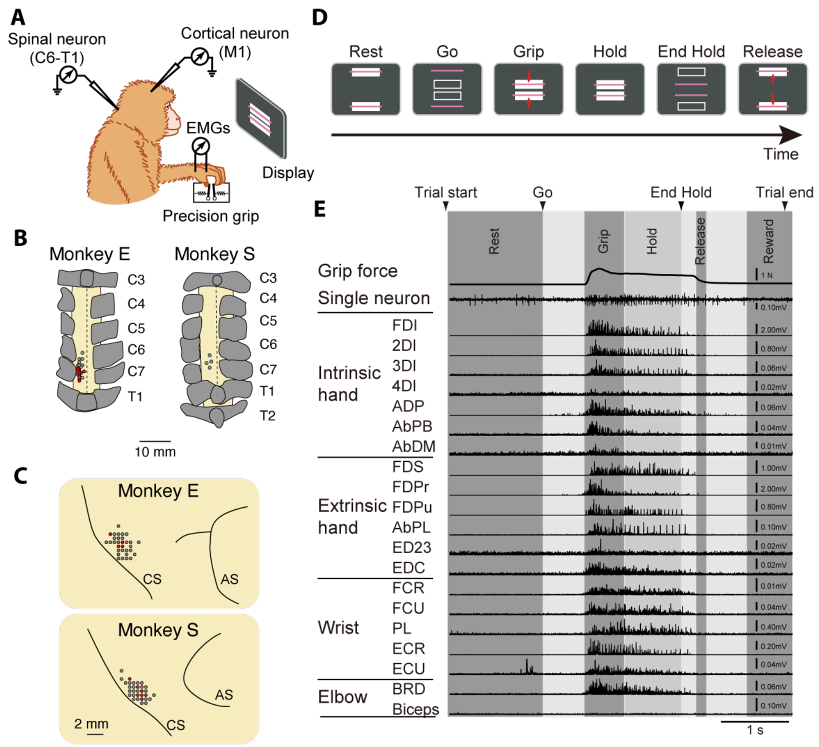

Fig. S1. Experimental setups

(**A**) Recording setup. We recorded single unit activity from spinal cervical cord or primary motor cortex and muscle activity of hand and arm muscles while the monkey was performing a precision grip task. (**B**) Spinal recording sites in each monkey. Grey dots indicate the locations where we recorded the spinal neurons. Red dots indicate the location where we recorded the identified PreM-INs or CM cells. Letters on the right indicate the number of vertebrae. Dorsal views are shown. (**C**) Cortical recording sites in each monkey. Grey and red dots in the same format as (**B**). CS, central sulcus; AS, arcuate sulcus. (**D**) Trial sequence. Monkeys were trained to grip, hold, and release the levers according to visual targets. (**E**) Example of single-unit activity (PreM-IN), muscle activity (n = 20), and grip force. FDI, first dorsal interosseous; 2DI, second dorsal interosseous; 3DI, third dorsal interosseous; 4DI, fourth dorsal interosseous; ADP, adductor pollicis; AbPB, abductor pollicis brevis; AbDM, abductor digiti minimi; FDS, flexor digitorum superficialis; FDPr, radial part of the flexor digitorum profundus; FDPu, ulnar part of the flexor digitorum profundus; AbPL, abductor pollicis longus; ED23, extensor digitorum-2,3; EDC, extensor digitorum communis; FCR, flexor carpi radialis; FCU, flexor carpi ulnaris; PL, palmaris longus; ECR, extensor carpi radialis; ECU, extensor carpi ulnaris; BRD, brachioradialis; Biceps, biceps brachii.

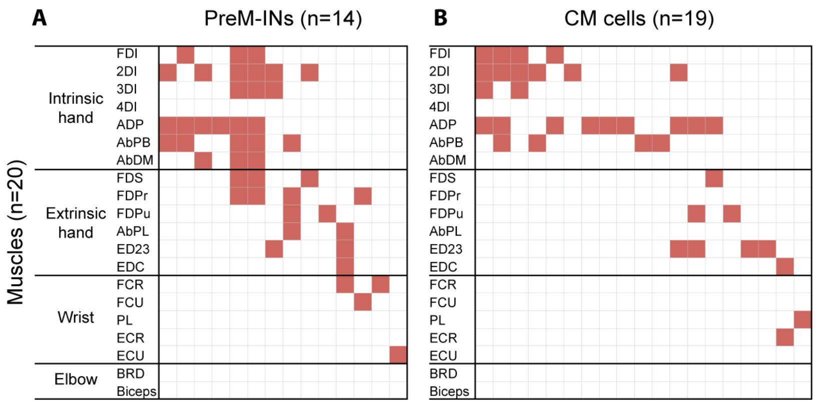

Fig. S2. Distribution of post-spike facilitations of PreM-INs and CM cells

Post-spike facilitation produced by 14 PreM-INs (**A**) and 19 CM cells (**B**). Each column and row indicates a neuron and a muscle, respectively. Red squares indicate significant post-spike facilitations.

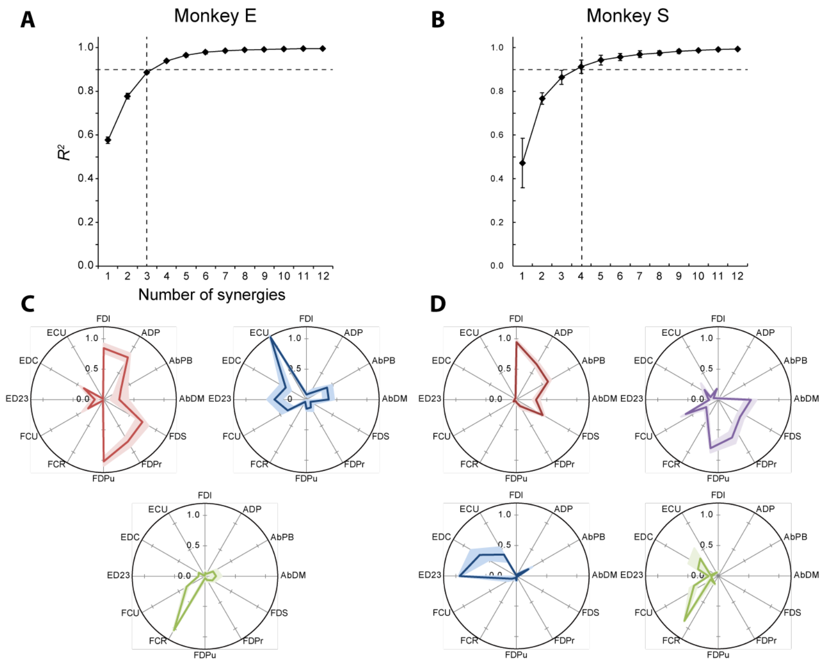

Fig. S3. Extraction of muscle synergies.

(**A–B**) Selection of the number of muscle synergies. Variance accounted for (R^2) as a function of the number of extracted synergies for monkeys E (**A**) and S (**B**). The R^2 curve was obtained by the fourfold cross-validation of the EMG data (filled circles). The number of muscle synergies (vertical dotted line) was selected where the R^2 was closest to 0.9 (horizontal dotted line). Error bars indicate SEM across sessions. (**C–D**) Muscle synergies extracted from monkeys E (**C**) and S (**D**). The radial axis indicates the weight of muscle synergy for each muscle, averaged across the fourfold cross-validation. Shaded areas indicate SEM across sessions.

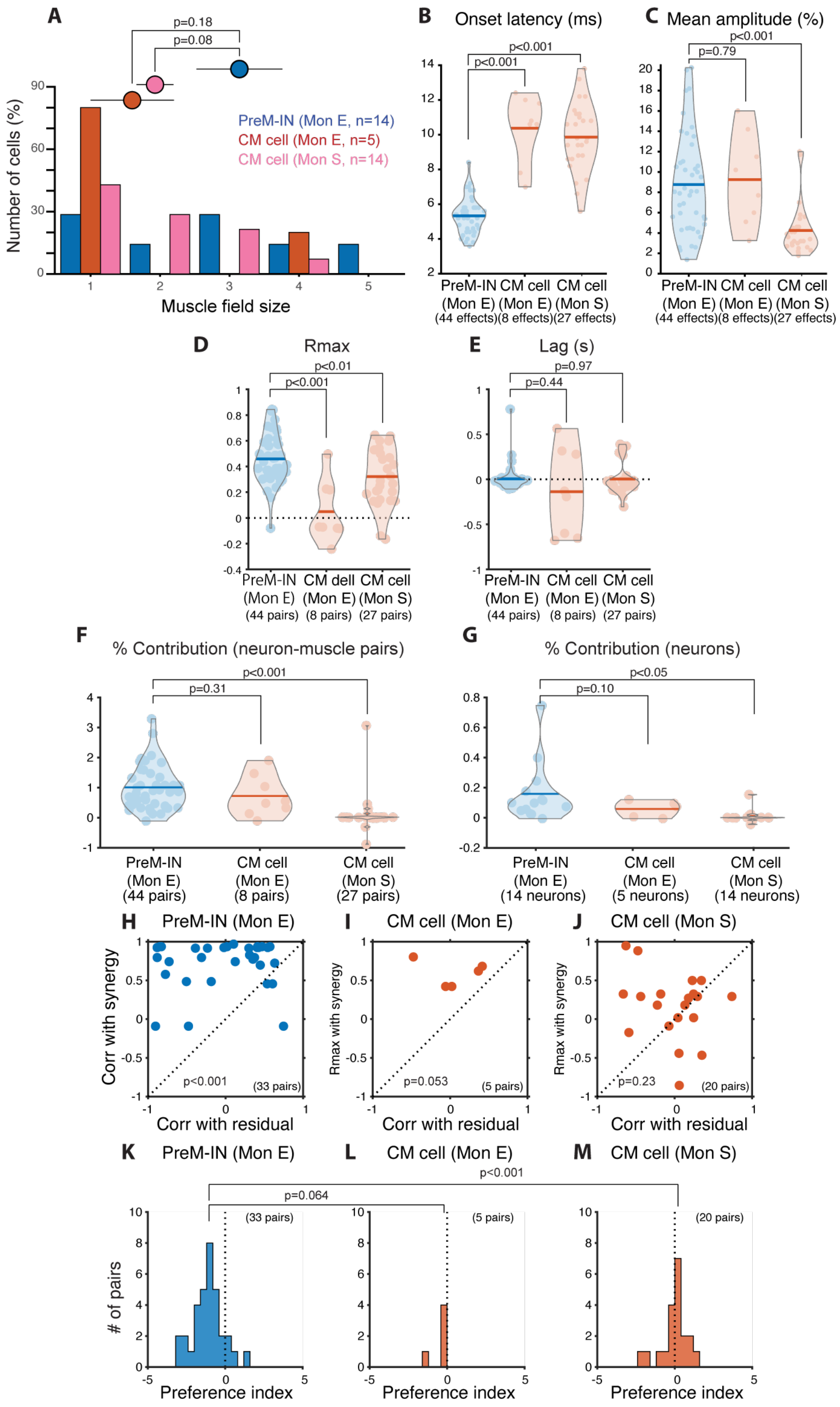

Fig. S4. Results separated for each animal

Data were compared between PreM-INs and CM cells of monkey E or that of monkey S. (**A**) Histogram of muscle field size. Same format as Fig. 1B. (**B–C**) Onset latency and mean amplitude of post-spike facilitations. Same format as Fig. 1C–D. (**D–E**) *Rmax* and lag of temporal correlation. Same format as Fig. 2G–H. (**F–G**) Percent contribution of neuron–muscle

pairs and neurons. Same format as Fig. 3D–E. (**H–J**) Correlation with the preferred synergy and residual of the target muscle. Same format as Fig. 4D. (**K–M**) Preference index. Same format as Fig. 4E.
